# An atypical case of intracerebral schwannoma

**DOI:** 10.11604/pamj.2014.18.342.5075

**Published:** 2014-08-27

**Authors:** Abdulrahman Abdullah AlBatly, Reem Seraj Zakzouk, Ali Khalaf Alhaidey

**Affiliations:** 1 Department of Radiology, Prince Sultan Medical Millitary City, Riyadh, Saudi Arabia

**Keywords:** Intracerebral schwannoma, immunohistochemically, glioblastoma multiforme

## Abstract

We report a case of intracerebral cystic schwannoma in the temporal fossa manifested as a gradually worsening headache in a 49-years-old woman. Computed Tomography (CT) and magnetic resonance imaging (MRI) showed a left temporal partly cystic, partly solid mass. The preoperative diagnosis was astrocytoma or glioblastoma multiforme (GBM), but microscopic examination of the mass showed the characteristic pattern with cellular Antony A component. Immunohistochemically, the tumor was positive for S-100 protein. These findings are consistent with a schwannoma. Intracerebral schwannomas not related to cranial nerves are rare and most reported cases involved young patients.

## Introduction

Intracranial schwannomas account for 8% of central nervous system tumors [1]. Most of these schwannomas arise from the vestibular portion of the VIIIth cranial nerve and less commonly, with descending order of frequency, from the Vth, IXth, Xth, and VIIth cranial nervesn [2], intraparenchymal schwannomas not related to cranial nerves are very rare, it represent less than 1%. To our knowledge, about 70 cases have been reported so far since the first case reported by Gibson *et al* in 1966 [3, 4].

## Patient and observation

A 49-year-old woman was admitted through the emergency department with a history of gradually worsening long standing headache and mild gait disturbance. There was no history of loss of consciousness, or visual deterioration. Neurological examination found mild right sided weakness and left 3rd nerve palsy. The rest of neurological examination was normal. The patient was admitted under neurosurgery. She experienced a single seizure episode during that time.

**Figure 1:**
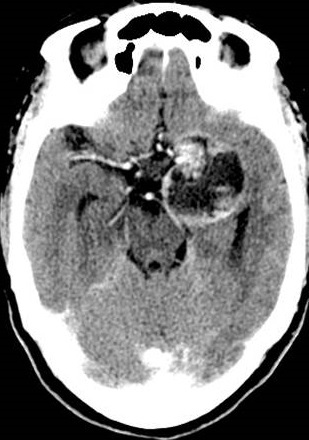
Axial Post enhanced CT scan shows left temporal lobe complex mass with peripheral thick nodular enhancement

**Figure 2:**
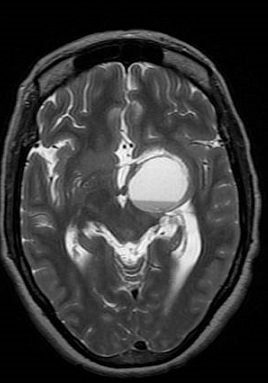
Axial T2WI shows fluid-fluid level within the mass and marked compression over the left cerebral peduncle of midbrain

**Figure 3:**
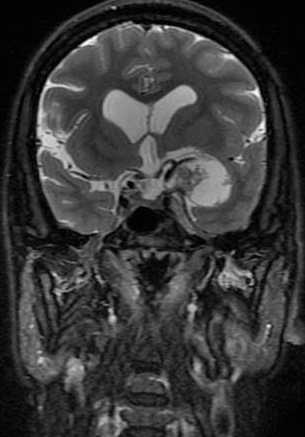
Coronal T2WI shows the mass surrounding the left MCA and mass effect over the left temporal horn of the lateral ventricle

**Figure 4:**
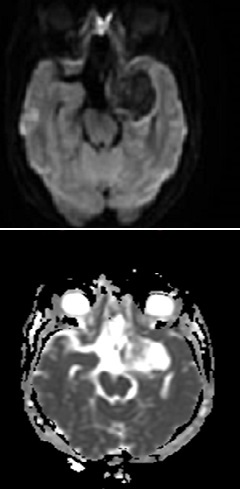
Diffusion-weighted image (DWI) and Apparent Diffusion Coefficient (ADC) map demonstrate no diffusion restriction

Computed Tomography (CT) and magnetic resonance imaging (MRI) showed intra-axial mass occupying the anteromedial aspect of left temporal lobe with a predominantly cystic component and peripheral solid nodules. Post contrast imaging showed peripheral thick nodular enhancement of the mass (Figure 1). Multiple fluid-fluid levels were noted within it likely due to hemorrhage of different ages (Figure 2). No appreciable perifocal edema or calcification. The lesion involved the left cavernous sinus with mass effect on the temporal horn of the left lateral ventricle and marked compression of the left cerebral peduncle of midbrain. It appeared to surround the M1 and M2 segments of the left middle cerebral artery (MCA) and is associated with mild midline shift to the right side (Figure 3). No diffusion restriction of the mass was seen (Figure 4). The preoperative diagnosis was astrocytoma or GBM.

The patient underwent a left fronto-temporal craniotomy with partial tumor removal. Only small fraction of the lesion that was adherent to the cavernous sinus was left behind. The pathological section confirmed a diagnosis of schwannoma. Patient did well in the immediate postoperative period with almost back to the preoperative neurological status.

Pathologic sections show a non-infiltrating, solid, spindle cell neoplasm and formation of patterning Verocay bodies with perivascular pseudo-rosettes, which is strongly S-100 positive but with nonspecific or weak GFAP reactivity. Ultrastructure demonstrates pericellular basal lamina collagen aggregates. The findings are consistent with those of cellular schwannoma.

## Discussion

Intracranial schwannomas account for 8% of central nervous system tumors [1]. Most of these schwannomas arise from the vestibular portion of the VIIIth cranial nerve. Intraparenchymal schwannomas not related to cranial nerves are very rare, it represent less than 1%. To our knowledge, about 70 cases have been reported so far.

Intraparenchymal schwannomas are located in both supratentorial and infratentorial areas; 63% and 37% respectively. The frontal and temporal lobes are the most common reported sites to be involved. They were reported to originate from the ventricular and periventricular structures. They have also been described in the cerebellar hemispheres,vermis and brain stem [4, 5].

Unlike vestibular schwannomas, the intraparenchymal schwannomas exhibit slight male predominance or even no female: male predominance. However Sharma *et al* reported a significant male to female ratio (M: F 3:1). Furthermore, the majority of cases have occurred in children and young adults, with average ages at presentation less than 21 years, unlike the vestibular schwannomas which usually occur in 5th decade unless associated with neurofibromatosis [6, 7].

The characteristic features of intraparenchymal schwannomas include calcification, cysts formation, peritumoral edema, gliosis and a solid enhancing component. A variety of contrast enhancement patterns were described, including those of a cyst with a mural nodule and peripheral enhancement [7]. According to Y. Haga *et al* in a review of 30 cases, calcification was found in 17%, cyst formation was found in 27%, and surrounding edema was found in 53% of the tumors. Khoo HM *et al* reported that calcification was noted in 29%, cyst formation in 68% and peritumoral edema in 87.5% of the cases. These characteristics appear to be more frequent in intracerebral schwannomas than in acoustic neurinomas and peripheral schwannomas [4].

However these findings are not specific and may be seen in other primary CNS tumors. Preoperative diagnosis is difficult. In fact, the preoperative diagnosis of our case was astrocytoma or GBM, and the final diagnosis of schwannoma was based on histological and immunochemistry examinations. In CT, these tumors show an isoattenuated or low-attenuation contrast-enhancing mass associated with a cystformation. The MRI characteristics are variable but usually they demonstrate a hypointense and hyperintense signal intensity on T1- and T2-weighted images, respectively. The salient radiological feature in the reported cases was a very frequent cystic component [5, 8, 9].

What make the presented case unique or unusual compared to the reviewed cases, is that it lacks calcification and peritumoral edema. The age also not typical, it is 49 years old which is much older than the mean age of reported cases in the literature. The mass showed fluid-fluid levels which indicate degradation of blood components, tied with only 1 other case reported by Casadei *et al* as frankly hemorrhagic lesion.

The histogenesis of intracranial schwannomas not arising from cranial nerves is still unclear. Since the Schwann cells are not normally present in the cerebral parenchyma, It is difficult to explain the origin of intraparenchymal brain schwannomas. Many theories have been proposed to explain the possible mechanism underlying the histogenesis and origin of these rare tumors. There are two common theories. One theory suggests a developmental origin, whereas the other suggests that they are more likely to be non-developmental in origin. According to the developmental theory, aberrant schwann cells in the brain parenchyma may occur due to the transformation of the mesenchymal pial cells, or from displaced neural crest cells that form the foci of schwann cells [2]. Non-developmental theories base their assumption on the fact that schwann cells have been detected around arteries in the intracranial perivascular nerve plexuses in the subarachnoid space and the brain parenchyma [10].

The radiological differential diagnosis of an intracerebral schwannoma includes many other neoplasms that may occur in children and young adults. These include piloctic astrocytoma, pleomorphic xanthoastrocytoma, ganglioglioma, meningioma and glioblastome multiforme. In order to differentiate between these tumors, exisional biopsy is usually needed. Further immunohistochemistry and/or electron microscopy are also needed to make the final diagnosis since some tumor like pilocytic astrocytoma and fibroblastic meningiomas resemble intracerebral schwannoma microscopically [11].

The clinical manifestations of intracerebral parenchymal schwannoma depend mainly on the locations and the sizes of the tumors. The most common symptoms and signs include headache as in our case, seizures, and focal neurological deficits. The treatment of choice for these tumors is total excision but this depends on the location of the tumors [12]. Complete relief of clinical symptoms and signs is mostly achieved after total or radical surgical removal [6].

The intracerebral schwannoma are benign tumors. Casadei *et al* and Sharma M C *et al* reported no evidence of tumor recurrence after complete resection in a follow-up period of 2-24 months. However, Stefanco *et al* had reported a highly malignant primary intracerebral schwannoma [13]. Also Singh RV *et al* had reported a case of malignant cerebellar schwannoma [14].

## Conclusion

The intracerebral schwannoma are almost benign tumors with very rare incidence. It may resemble the astrocytoma or GBM. The treatment of choice for these tumors is total excision with complete relief of clinical symptoms and signs mostly achieved.

## References

[1] Lin J, Feng H, Li F (2003). Intraparenchymal schwannoma of the medulla oblongata: case report. J Neurosurg..

[2] Russels DS, Rubinstein LJ (1989). Pathology of tumors of the nervous system.

[3] Luo W, Ren X, Chen S, Liu H, Sui D, Lin S (2013). Intracranial intraparenchymal and intraventricular schwannomas: report of 18 cases. Clin Neurol Neurosurg..

[4] Hui Ming Khoo, Takuyu Taki (2012). Periventricular intraparenchymal schwannoma: Case report. Neurol Med Chir (Tokyo).

[5] Casadei GP, Komori T, Scheithauer BW, Miller GM, Parisi JE, Kelly PJ (1993). Intracranial parenchymal schwannoma: a clinicopathological and neuroimaging study of nine cases. J Neurosurg..

[6] Sharma MC, Karak AK, Gaikwad SB, Mahapatra AK, Mehta VS, Sudha K (1996). Intracranial intraparenchymal schwannomas: a series of eight cases. J Neurol Neurosurg Psychiatry..

[7] Zagardo MT, Castellani RJ, Rees JH (1998). Radiologic and pathologic findings of intracerebral schwannoma. AJNR Am J Neuroradiol..

[8] Luo B, Sun G, Zhang B, Liang K, Wen J, Fang K (2004). Neuroradiological findings of intracranial schwannomas not arising from the stems of cranial nerves. The British Journal of Radiology..

[9] Muzzafar S, Ketonen L, Weinberg JS, Schellingerhout D (2010). Imaging and Clinical Features of an Intra-Axial Brain Stem Schwannoma. AJNR Am J Neuroradiol..

[10] Nelson E, Rennels M (1970). Innervation of intracranial arteries. Brain..

[11] Sobel RA, Michaud J (1985). Microcystic meningioma of the falx cerebri with numerous palisading structures: an unusual histological pattern mimicking schwannoma. Acta Neuropathologica..

[12] Sharma RR, Gurusinghe NT, Lynch PG (1993). Intraparenchymatous schwannoma of the cerebellum. Br Neurosurg..

[13] Stefanko SZ, Vuzevski VD, Maas AI, van Vroonhoven CC (1986). Intracerebral malignant schwannoma. Acta Neuropathol..

[14] Singh RV, Suys S, Campbell DA, Broome JC (1993). Malignant schwannoma of the cerebellum: case report. Surg Neurol..

